# Waist-Stature Ratio And Its Relationship With Autonomic Recovery From Aerobic Exercise In Healthy Men

**DOI:** 10.1038/s41598-018-34246-5

**Published:** 2018-10-31

**Authors:** Anne Michelli G. G. Fontes, Letícia S. de Oliveira, Franciele M. Vanderlei, David M. Garner, Vitor E. Valenti

**Affiliations:** 1 Post-Graduate Program in Physical Therapy, UNESP, Presidente Prudente, SP Brazil; 2 Cardiorespiratory Research Group, Department of Biological and Medical Sciences, Faculty of Health and Life Sciences, Oxford Brookes University, Gipsy Lane, Oxford, OX3 0BP United Kingdom; 3 Autonomic Nervous System Center (CESNA), UNESP, Marilia, SP Brazil

## Abstract

Autonomic modulation and cardiorespiratory variables are influenced by numerous factors, including anthropometric variables. We investigated autonomic recovery following aerobic exercise in healthy men with different waist-stature ratio (WSR) values. The study was conducted with 52 healthy men aged 18 to 30 years, divided into groups according to the WSR: G1 – between 0.40 and 0.449 (N = 19), G2 – between 0.45 and 0.50 (N = 28) and G3 – between 0.5 and 0.56 (N = 5). The subjects endured 15 minutes seated and at rest followed by an aerobic exercise and then remained seated for 60 minutes and at rest during recovery from exercise. Heart rate (HR) variability (HRV) (rMSSD, SD1, HF [ms^2^]) and cardiorespiratory variables were analyzed before and after exercise. Recovery of respiratory rate, diastolic blood pressure, SD1 and HF indices were delayed in G2. G3 presented delayed recovery after the maximal effort test while no difference with G2 was noted in the moderate intensity. Correlation and linear regression analysis indicated association of WSR, body mass index and waist circumference with HRV indices in the recovery from aerobic exercise (45 to 60 minutes after exercise) in G2. In conclusion, healthy men with higher WSR accomplished delayed autonomic recovery following maximal effort exercise.

## Introduction

Previous studies have revealed that excessive adiposity in the abdominal region is related to various metabolic and cardiovascular dysfunctions, such as hypertension, diabetes mellitus and dyslipidemia, and these are linked with increased risk of morbidity and mortality^1–3^.

Amongst the indicators of abdominal obesity, body mass index (BMI), waist circumference (WC), hip circumference (HC), conicity index (CI), waist-stature ratio (WSR) have been studied and are widely accepted in disease assessment, management and predictions in clinical practice and public health surveillance^4,5^.

Lately, WSR has been widely applied as it is simple, easy to measure and calculate. It is obtained by dividing the WC by height, in which WC demonstrates abdominal obesity and height remains constant in adults, which allows the possibility for direct comparisons in the general population^6,7^.

The research literature has testified that this relationship is an adequate predictor of metabolic and cardiovascular risk^8–10^, which reinforces its implementation in clinical practice. Moreover, this measure has also been associated with changes in the autonomic balance. Indumathy *et al*.^11^ investigated the association of the anthropometric indices and cardiovascular parameters with the autonomic nervous system (ANS) in 88 individuals split into control and obese groups submitted to electrocardiographic recording and autonomic tests. These researchers observed that amongst the anthropometric indices, WC, WHR and WSR were connected to sympathetic and parasympathetic activity.

In this context, one way to analyze the ANS activity is through heart rate variability (HRV), a non-invasive technique that describes the fluctuations in the intervals between consecutive heart beats (RR intervals). These are linked to the influence of the ANS on the sinoatrial (SA) node^12,13^.

In addition to HRV, another indicator of autonomic functioning is heart rate recovery following exercise. This is controlled by vagal reactivation and sympathetic withdrawal and is considered a good predictor of mortality related to cardiovascular disease and other pathologies in the general population^14^.

Although the research literature has focused on obese patients with increased risk based on the WSR^4,5^, it lacks evidence in subjects with values closer to the limit, herein moderate risk. In this sense, we appraised autonomic recovery after aerobic exercise in healthy men with different ranges of WSR.

## Methods

### Population

We investigated 52 healthy men with a mean age of 22.15 ± 2.68 years. All were physically active according to the International Physical Activity Questionnaire (IPAQ)^15^ and recruited in the Faculty of Philosophy and Sciences of the Paulista State University, Marilia, São Paulo, Brazil.

We excluded individuals with cardiorespiratory, neurological, musculoskeletal, renal, metabolic, endocrine and other reported impairments that circumvented the satisfactory performance of the procedures. Individuals with systolic blood pressure (SBP) greater than 130 mmHg and diastolic blood pressure (DBP) more than 90 mmHg at rest, smokers, sedentary and insufficiently active individuals according to IPAQ were also omitted.

We excepted subjects who did not complete all stages of the experimental protocol and then those classified as obese according to the studies of Lohman *et al*.^16^.

Subjects were split according to WSR: G1 – between 0.40 and 0.449 (N = 19), and G2 – between 0.45 and 0.49 (N = 28), as these values link to moderate risk of developing cardiovascular disease^17^. In order to enhance data in the moderate risk subjects we also evaluated the excluded subjects with WSR between 0.5 and 0.56 (G3, N = 5). G3 was composed by a small sample because we focused on moderate risk groups (G1 and G2).

### Ethical approval and informed consent

All subjects were informed about procedures and objectives of the study and, after agreeing, signed an informed consent letter. The project was approved by the Research Ethics Committee of the Faculty of Philosophy and Sciences of the Paulista State University, Marília, São Paulo, Brazil (Number 5406). All methods were performed in accordance with the 466/2012 National Health Council Resolution of 10/10/1996.

### Initial assessment

The introductory evaluation was commenced to investigate the eligibility criteria and to obtain descriptive statistical characterizations about the individuals. An anamnesis was completed to confirm the absence of recognized diseases and medication use, followed by the application of the IPAQ questionnaire to investigate the level of physical activity and the willingness to participate in the experimental procedure.

The anthropometric measurements were attained according to the recommendations described by Lohman *et al*.^16^. Mass was measured via a digital scale (W200/5, Welmy, Brazil) with a precision of 0.1 kg. The height was determined on a stadiometer (ES2020, Sanny, Brazil) with an accuracy of 0.1 cm. The BMI was calculated via the following formula: mass (kg)/height (m)^2^.

The measurement of WC was made in orthostatism, with the abdomen relaxed and arms extended along the body, being recorded with a measuring tape positioned in the area of lesser curvature located between the last rib and the iliac crest. The WSR was calculated using the following formula: WC (cm)/height (cm).

HR was assessed via the Polar RS800cx HR monitor (Polar Electro, Finland) and respiratory rate (F) was measured by counting the respiratory cycles during one minute whilst the volunteer was unaware of the process thus avoiding influences and consequent changes in the subjects’ respiratory patterns.

Blood pressure was calculated indirectly by auscultation using a calibrated aneroid sphygmomanometer and stethoscope (Premium, Barueri, São Paulo, Brazil) on the left arm while the individual remained seated and breathing spontaneously.

To avoid misrepresentations in the measurements, the same researcher measured the same parameters throughout the experiment.

### Protocol

The data collection was performed individually between 17:00 and 22:00 in a soundproofed room with controlled temperature between 21 °C and 25 °C and humidity between 50% and 60% at the Faculty of Philosophy and Sciences of the Paulista State University, Marília, Brazil.

The subjects were instructed to avoid drinking alcoholic beverages for 24 hours prior to the evaluation, food or caffeinated drinks 8 hours before the assessment. Similarly, not to perform strenuous exercises for 24 hours before appraisal, to consume only a light meal 2 hour before and to wear appropriate and comfortable clothing to undergo the necessary physical exertion.

After the preliminary evaluation, the HR Polar RS800cx receiver (Polar Electro, Finland) was positioned on the subjects’ thorax, at the level of the distal third of the sternum. The procedure was comprised of two sessions, all performed on a treadmill, with a minimum interval of 48 hours, to allow adequate recovery of the subjects. The following protocols were undertaken:

1. Maximum effort test protocol^18^: performed to regulate the maximum velocity (V_max_) through the Conconi Threshold and the subsequent use of 60% of this value for the load applied in the following step.
2. Aerobic exercise protocol^18^: rest for 15 minutes in the seated position under natural breathing, followed by 5 initial minutes with velocity of 6.0 km/h + 1% inclination for physical ‘warming up’, afterwards 25 minutes with intensity equivalent to 60% of the V_max_ + 1% of inclination, and to conclude 60 minutes of recovery^19^.

In both protocols, immediately after the exercise, subjects endured three minutes standing and subsequently seated for passive recovery for a further 57 continuous minutes, totaling 60 minutes of recovery. Throughout recovery from exercise volunteers remained seated in silence under spontaneous breathing, they did not sleep, did not perform any movement that induce autonomic changes and did not ingest any type of drink or food.

In the maximum effort test protocol, HR and HRV indices were analyzed at the following times: Rest (10^th^ to 15^th^ minute of resting) and during recovery (Rec): Rec1 (5^th^ to 10^th^ minute), Rec2 (15^th^ to 20^th^ minute), Rec3 (25^th^ to 30^th^ minute), Rec4 (35^th^ to 40^th^ minute), Rec5 (45^th^ to 50^th^ minute) and Rec6 (55^th^ to 60^th^ minute)^18^.

In the aerobic exercise protocol, F, HR, SBP and DBP were recorded at 15 minutes of rest and at 1, 3, 5, 7, 10, 20, 30, 40, 50 and 60 minutes during recovery from exercise^18^. HRV analysis was finalized at the following times: Rest (10^th^ to 15^th^ minute of resting) and during recovery (Rec): Rec1 (5^th^ to10^th^ minute), Rec2 (15^th^ to 20^th^ minute), Rec3 (25^th^ to 30^th^ minute), Rec4 (35^th^ to 40^th^ minute), Rec5 (45^th^to 50^th^ minute) and Rec6 (55^th^ to 60^th^ minute)^18^.

### Assessment of maximum aerobic capacity and power

A maximum effort test was commenced to determine the V_max_ through the Conconi threshold and was proposed for the indirect assessment of the anaerobic threshold via identification of the HR deflection point (HRDP) using a progressive test using the D_max_ method^20^. The volunteers experienced a comprehensive progressive treadmill test (Evolution Fitness, EVO 4000) with an initial velocity of 8 km/h and load increments of 1 km/h every 2 minutes until exhaustion^18,19,21^. To be approved as the maximum test, the subjects reached 90% of their maximum HR (HR_max_) computed by the 220 - age formula^22^ and the perception of effort was recorded according to the Borg scale^23^.

For the identification of HRDP, the HR points and their corresponding velocities were plotted. Next, the values were adjusted by means of a first-degree linear equation and a third-degree polynomial function derived from the individuals’ data. Then, the difference of the HR values obtained by the respective equations was calculated and when generating a curve with these values, the highest value was nominated HRDP before a transformation in the direction of the curve^20^.

The value of HRDP corresponds to the speed at which the volunteer attained their anaerobic threshold. This value was compared to the value of 60% of the V_max_ realized in the exercise test and for use of the intensity in the succeeding stage, this should be inferior to that accomplished in the anaerobic threshold.

### HRV analysis

HR was recorded beat-to-beat throughout with the HR monitor (Polar RS800cx, Finland) and RR intervals recorded by the cardiac portable monitor. These were transferred to the Polar Pro Trainer program (v.3.0, Polar Electro, Finland). Digital filtering accompanied with manual filtering was completed for the disposal of artifacts.

For data analysis, appropriate RR intervals were selected for analysis and extracted into a ‘txt’ format. All indices were evaluated using a fixed number of 256 consecutive stable RR intervals obtained from the baseline ending as well as the final 256 intervals of each recovery period. Only series with more than 95% of sinus beats were included in the study.

This counting method for RR intervals followed directives from the Task Force^12,24^ and were before now published in previous studies^25–28^.

Kubios HRV^®^ software (Kubios^®^ HRV v.1.1 for Windows, Biomedical Signal Analysis Group, Department of Applied Physics, University of Kuopio, Finland) was used to compute linear indices.

For the analysis in the frequency domain, the high frequency spectral component (HF) was used in absolute units (ms^2^). The time domain analysis was undertaken via the rMSSD indices (square root of the mean of the square of the differences between adjacent normal RR intervals in a time interval expressed in milliseconds (ms), and SD1 (instantaneous variability of beat-to- beating)^29^.

### Statistical analysis

The sample size was attained by the calculation based on a pilot test, wherein the online software provided by the website www.lee.dante.br was required taking into consideration the rMSSD index as a variable. The significant difference in magnitude assumed was 14.11 ms, with a standard deviation of 12.8 ms, per alpha risk of 5% and beta of 80%, the sample size determined was a minimum of 13 individuals per group.

For data analysis, descriptive statistics were necessary to characterize the sample and the results were described by the statistical values of mean, median, standard deviation, minimum and maximum values.

The HRV indices’ assessments and those of the cardiorespiratory variables between groups and moments (rest vs. moments of recovery) were performed via the two-way repeated measures analysis of variance technique (ANOVA2). The data of the repeated measurements were checked for sphericity violation using the Mauchly test and the Greenhouse-Geisser correction was applied when the sphericity was violated.

The normality of the data was determined using the Shapiro-Wilk test. Based on these test results, the unpaired Student t test (parametric) or the Mann-Whitney test (non-parametric) were required to compare descriptive characteristics between groups.

In order to compare variables between groups we applied two way ANOVA (group vs. WSR) followed by Bonferroni posttest, since all data were parametric distributions.

For analysis of the moments (rest vs. recovery times), the repeated measurements one-way analysis of variance (ANOVA1) test followed by Dunnett’s test (parametric distribution) or the Friedman test followed by Dunn’s test (non-parametric distribution) were enforced.

In order to evaluate the association between HRV indices and anthropometric variables (BMI, WC and WSR) we used the Pearson correlation coefficient test for parametric distributions and the Spearman correlation coefficient test was applied for non-parametric distributions. We considered strong correlations for r > 0.75, moderate correlations for r between 0.5 and 0.75 and weak correlation for r < 0.5.

With the intention to investigate the effect of independent variables on dependent variables a simple linear regression model was constructed. The selection of the independent variables was achieved initially by the correlation analysis, taking into consideration only the variables with a significant correlation (p < 0.05). A simple linear regression model was required to model the HRV indices as outcome variables. Predictors included continuous variables representing BMI, WC and WSR. The R^2^ was estimated to verify the coefficient of determination of the percentage of variation explained by the model. The adjusted-R^2^ was obligatory to evaluate the stability of the model.

Modifications in all tests were considered statistically significant when the p-value was less than 0.05 (or < 5%).

The computations were performed via Minitab software (Minitab, PA, USA), GraphPad InStat - v 3.06 (GraphPad Software, Inc., San Diego California USA) and IBM SPSS Statistics - version 22.0 (SPSS Inc. Chicago USA).

## Results

The descriptive statistics of the sample are presented in Table 1, wherein the similarity between the groups in relation to the described variables is expressed, with the exclusion of mass, BMI, WSR and WC, which were higher in G2 compared to G1 and higher in G3 compared to G1 and G2.

**Table 1:** Mean values followed by their respective standard deviations, median, minimum and maximum of age, mass, height, BMI, WSR, WC, HR_max_, Conconi threshold, peak velocity and 60% velocity. ^*^Difference between G1 and G2; ^**^Difference between G1 and G3; ^***^Difference between G2 and G3.

Variables	G1	G2	G3	p	F
Age (Years)	22.00 ± 2.35 (23.00) [18.00–28.00]	22.31 ± 3.04 (21.50) [19.00–28.00]	23.8 ± 3.27 (25) [19–27]	0.388	0.961
Mass (Kg)	69.04 ± 8.78 (68.70) [54.00–84.00]	76.49 ± 7.62 (75.50) [64.80–93.70]	86.44 ± (87.3) [75–100.4]	*^,^ **0.0001	10.483
Height (m)	1.76 ± 0.06 (1.75) [1.66–1.90]	1.75 ± 0.05 (1.75) [1.62–1.89]	1.73 ± 0.08 (1.69) [1.64–1.86]	0.52	0.65
BMI (Kg/m^2^)	22.18 ± 2.33 (22.12) [18.46–26.65]	24.80 ± 1.54 (24.59) [22.19–29.24]	28.8 ± 2.37 (28.64) [26.26–32.6]	*^,^**^,^***<0.0001	22.009
WC (m)	0.76 ± 0.04 (0.75) [0.68–0.83]	0.83 ± 0.02 (0.82) [0.78–0.88]	0.92 ± 0.01 (0.92) [0.9–0.94]	*^,^**^,^***<0.0001	26.723
WSR	0.42 ± 0.018 (0.43) [0.38–0.448]	0.47 ± 0.0117 (0.47) [0.45–0.49]	0.53 ± 0.02 (0.532) [0.5–0.56]	*^,^**^,^***<0.0001	54.614
HR_max_	198.15 ± 2.4 (198) [192–202]	197.65 ± 2.8 (197.5) [192–201]	196 ± 3.2 (195) [193–201]	0.428	0.8615
Conconi threshold	11.02 ± 0.84 (11) [9.44–12.45]	11.16 ± 1.74 (10.98) [6.83–16.38]	10.49 ± 0.06 (10.49) [10.44–10.539]	0.52	0.6466
Peak velocity	13.15 ± 1.21 (13) [11–15]	13.27 ± 1.82 (13) [11–18]	12 ± 0 (12) [12–12]	0.448	0.817
60% velocity	7.89 ± 0.72 (7.8) [6.6–9]	7.96 ± 1.09 (7.8) [6.6–10.8]	7.2 ± 0 (7.2) [7.2–7.2]		

BMI: body mass index; WC: waist circumference; WSR: waist-stature ratio; HR_max_: maximum heart rate; G1: group with WSR between 0.40 and 0.449; G2: group with WSR between 0.45 and 0.49; G3: group with WSR between 0.5 and 0.56; kg: kilogram; m: meters.

In order to compare details regarding exercise protocol we compared maximum HR, Conconi threshold, peak velocity and 60% velocity between G1, G2 and G3 (Table 1). We noted no significant differences between groups.

**Figure 1:**
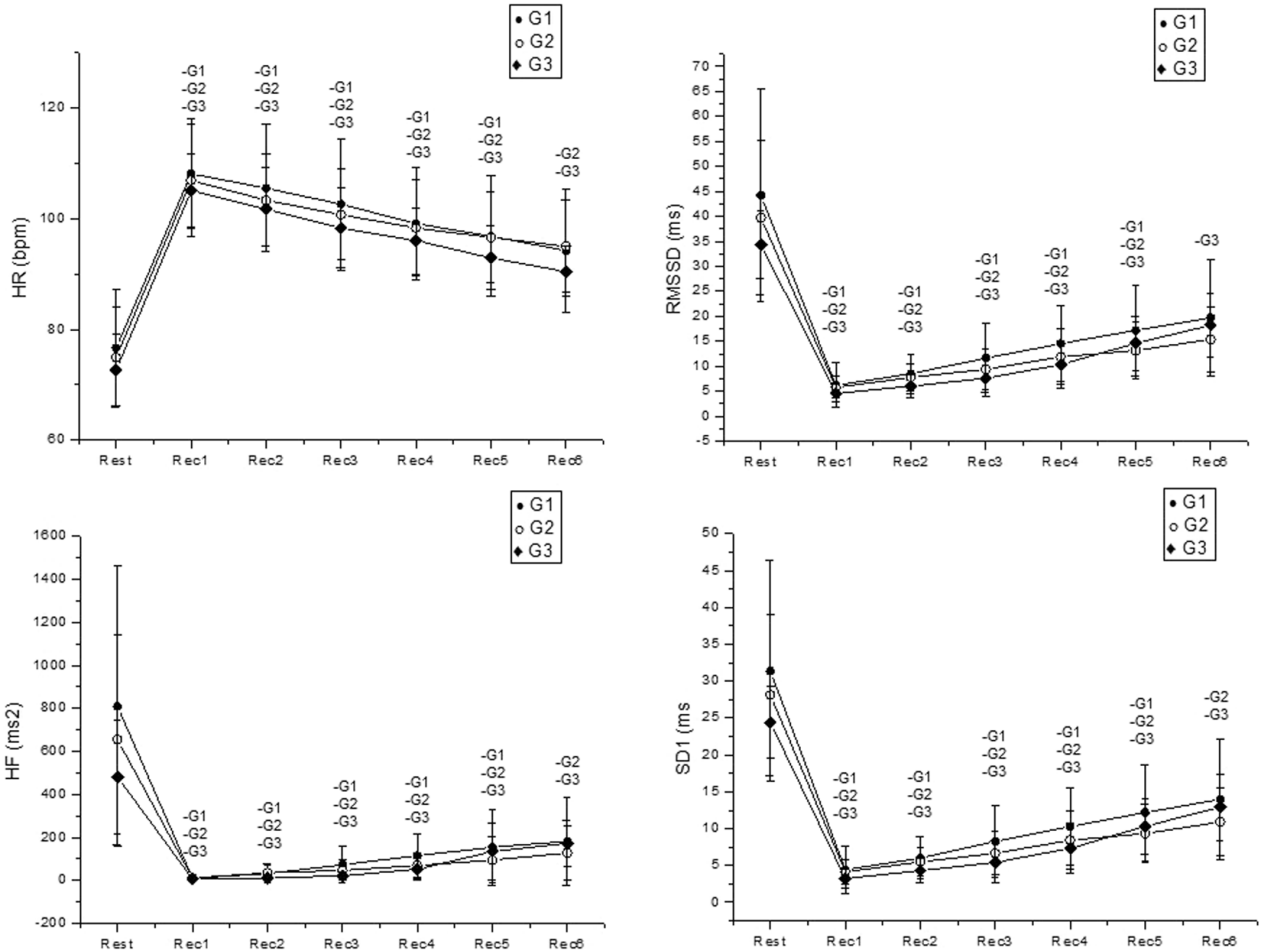
Mean values and respective standard deviations of HR, rMSSD, HF and SD1 indexes obtained at rest and during recovery from the maximal test effort. -G1, -G2 and -G3: Values with significant differences in relation to rest (p < 0.0001). HR: heart rate; rMSSD: square root of the square mean of the differences between adjacent normal RR intervals; SD1: standard deviation of instantaneous beat-to-beat variability; HF: high frequency; G1: group with WSR between 0.40 and 0.449; G2: group with WSR between 0.45 and 0.49; G3: group with WSR between 0.5 and 0.56; bpm: beats per minute; ms: milliseconds.

We observed that during the maximal effort test there was an effect of the moment for all variables analyzed (p < 0.0001). No effect was observed between groups for all indices: Mean HR (p = 0.91), rMSSD (p = 0.23), HF [ms^2^] (p = 0.29) and SD1 (p = 0.23), as well as no moment interaction and groups for all indices analyzed (Mean HR, p = 0.56; rMSSD, p = 0.57, HF [ms^2^], p = 0.63, SD1, p = 0.55) (Fig. 1).

In relation to the maximum effort, for the rMSSD, HF and SD1 indices in G1, a significant reduction was observed when the rest was compared with the moments from Rec 1 to Rec 5. For Mean HR, SD1 and HF [ms^2^] the difference was between the basal moment compared to all other moments of the recovery in G2. rMSSD was different when the rest was compared with the moments from Rec 1 to Rec 5 in G2. For Mean HR there was an increase in the values and for the HF index [ms^2^] there was a decrease in the values when compared to the initial rest time. We also noted significant difference between the basal moment compared to all other moments of the recovery in G3 (Fig. 1).

**Figure 2:**
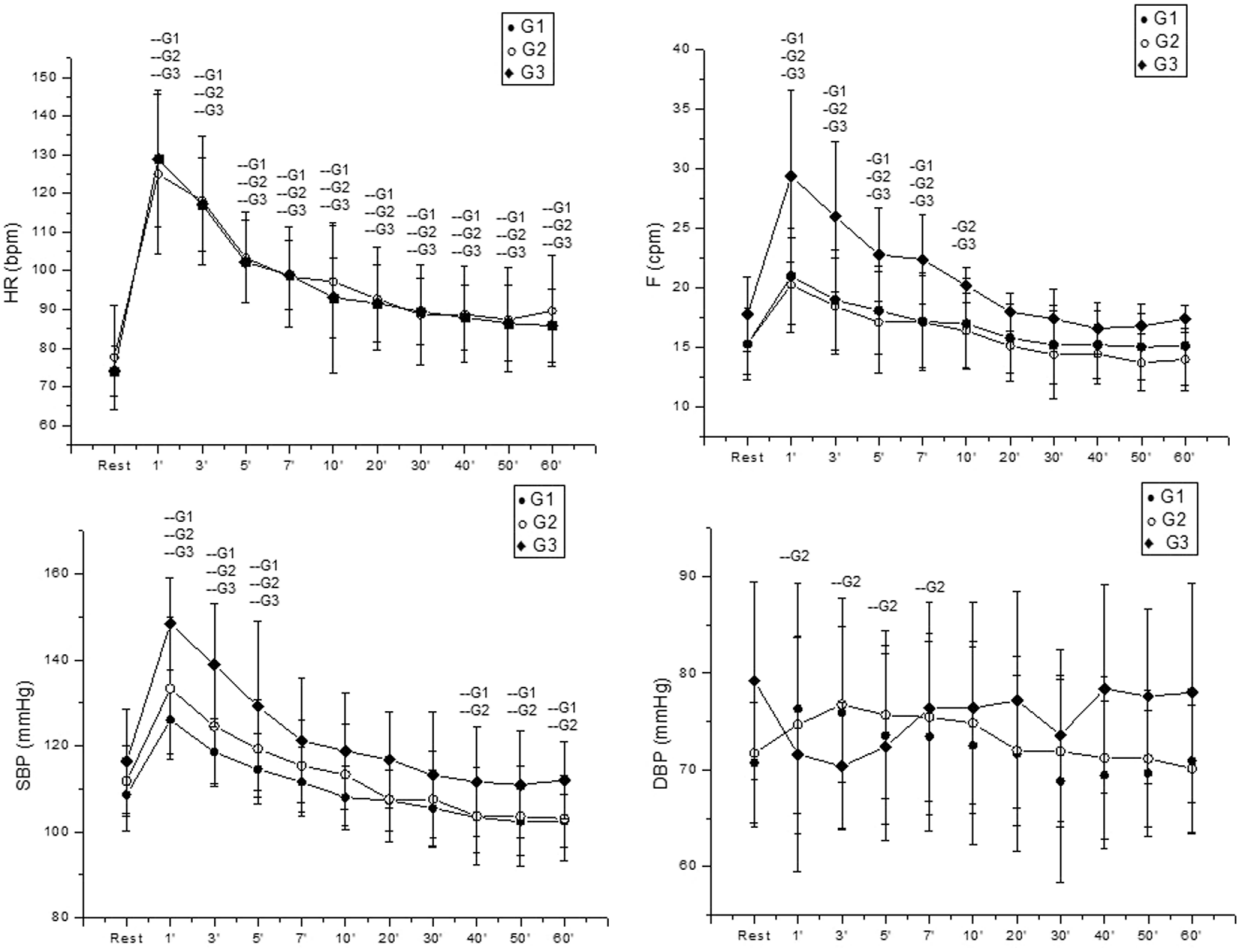
Mean values and respective standard deviations of HR, F, SBP, DBP obtained at rest and during recovery from moderate aerobic exercise protocol. -G1, -G2 and -G3: Values with significant differences in relation to rest (p < 0.01). SBP: systolic blood pressure; DBP: diastolic blood pressure; HR: heart rate; F: respiratory rate; G1: group with WSR between 0.40 and 0.449; G2: group with WSR between 0.45 and 0.49; G3: group with WSR between 0.5 and 0.56; mmHg: millimeters of mercury; bpm: beats per minute; rpm: breaths per minute.

Cardiorespiratory variables during rest and recovery after exercise is demonstrated in Fig. 2. A significant effect of time in HR, F, SBP and DBP in G1 and G2 (p < 0.0001) is observed.

No effects were observed between the groups (HR: p = 0.57; F: p = 0.23; SBP: p = 0.45; DBP p = 0.999) and interaction between moments vs. groups (HR: p = 0.68; F: p = 0.54, SBP: p = 0.1, DBP: p = 0.65) (Fig. 2).

Regarding HR response, a recovery after 60 minutes was realized for all groups, in relation to F the G1 recovered after the 7^th^ minute post-exercise while G2 and G3 recovered after the 10^th^ minute post-exercise (Fig. 2).

Regarding SBP values, all groups presented a recovery after the 5^th^ minute, whereas for DBP there was no significant difference between rest and post-exercise in the G1 and G3 whereas the G2 recovered after the 10^th^ minute post-exercise.

**Figure 3:**
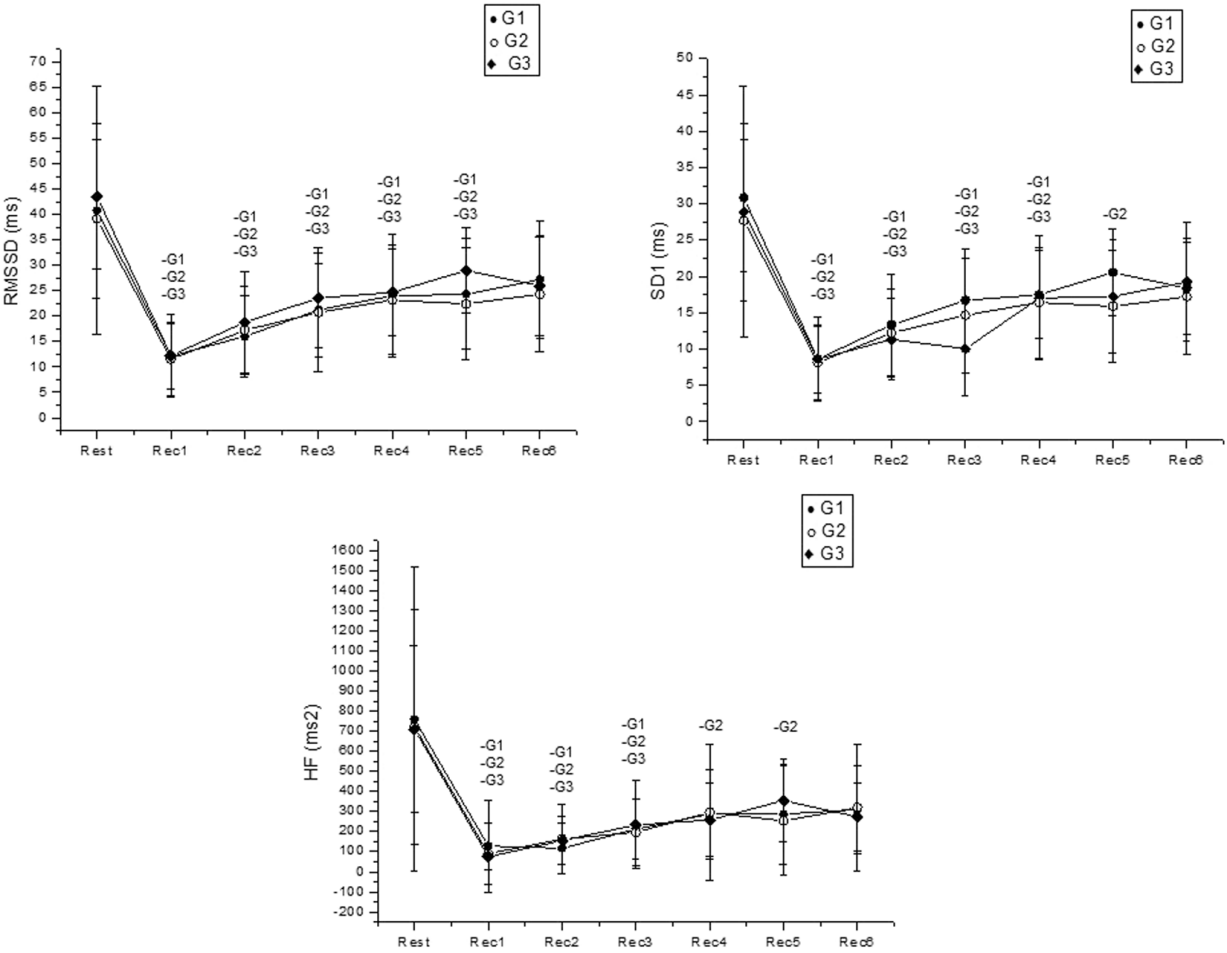
Mean values and respective standard deviations of rMSSD, SD1 and HF [ms^2^] indexes obtained at rest and during recovery from moderate aerobic exercise protocol. -G1, -G2 and -G3: Values with significant differences in relation to rest (p < 0.001). rMSSD: square root of the square mean of the differences between adjacent normal RR intervals; SD1: standard deviation of instantaneous beat-to-beat variability; HF: high frequency; G1: group with WSR between 0.40 and 0.449; G2: group with WSR between 0.45 and 0.49; G3: group with WSR between 0.5 and 0.56; ms: milliseconds.

Figure 3 displays HRV indices during the recovery from the moderate aerobic exercise and its comparison with resting values. We observed a moment effect for rMSSD, SD1 and HF [ms^2^] indexes (p < 0.0001), yet, no effects were detected between groups (rMSSD: p = 0.54; SD1: p = 0.56; HF [ms^2^]: p = 0.3) and in the interaction between moments vs. groups (rMSSD: p = 0.54; SD1: p = 0.56; HF [ms^2^]: p = 0.5).

The rMSSD index recovered after Rec5 for all groups. The SD1 index improved after Rec4 in the G1 and G3 and after Rec5 in the G2 group while the HF index [ms^2^] similarly recovered after Rec3 in the G1 and G3 and after Rec5 in the G2 group.

We performed a correlation analysis to verify whether there is an association between anthropometric variables and HRV. Table 2 shows no significant correlation between the aforementioned variables in G1.

**Table 2:** Correlation between anthropometric variables and HRV indices G1 in the moderate aerobic exercise protocol in. mmHg: millimeters of mercury. m: meters; kg: kilograms; mmHg: millimeters of mercury.

G1	Rest		Rec 1		Rec 2		Rec 3		Rec 4		Rec 5		Rec 6	
BMI	r	p	r	p	r	p	r	p	r	p	r	p	r	p
rMSSD	−0.1	0.45	−0.09	0.69	−0.17	0.46	−0.06	0.78	−0.12	0.62	−0.07	0.74	0.09	0.7
HF	0.14	0.55	−0.13	0.59	−0.06	0.79	−0.02	0.93	0.02	0.91	0.41	0.07	−0.08	0.72
SD1	0.18	0.46	−0.09	0.69	−0.18	0.45	−0.06	0.78	−0.12	0.61	−0.07	0.74	0.09	0.7
**WC**	**r**	**p**	**r**	**p**	**r**	**p**	**r**	**p**	**r**	**p**	**r**	**p**	**r**	**p**
rMSSD	0.01	0.95	0.25	0.28	0.31	0.18	0.21	0.38	0.16	0.49	0.15	0.53	0.27	0.24
HF	−0.01	0.94	0.31	0.19	0.44	0.05	0.18	0.45	0.27	0.25	0.24	0.32	0.24	0.3
SD1	0.1	0.95	0.25	0.28	0.31	0.19	0.2	0.38	0.16	0.49	0.15	0.53	0.28	0.24
**WSR**	**r**	**p**	**r**	**p**	**r**	**p**	**r**	**p**	**r**	**p**	**r**	**p**	**r**	**p**
rMSSD	0.08	0.81	−0.06	0.8	−0.06	0.8	0.006	0.97	−0.01	0.94	−0.02	0.91	0.12	0.6
HF	0.12	0.62	−0.02	0.9	0.007	0.97	−0.01	0.95	0.09	0.71	0.22	0.35	0.24	0.31
SD1	0.05	0.81	−0.06	0.8	−0.06	0.79	0.006	0.98	−0.01	0.94	−0.02	0.9	0.12	0.6

BMI: body mass index; WC: waist circumference; WSR: waist-stature ratio; HR: heart rate; rMSSD: square root of the square mean of the differences between adjacent normal RR intervals; SD1: standard deviation of instantaneous beat-to-beat variability; HF: high frequency; G1: group with WSR between 0.40 and 0.449.

Then again, we demonstrated an interaction in G2. rMSSD and SD1 presented significant negative weak correlation with BMI at Rec 4 and Rec 5 and significant negative moderate correlation in Rec 6. There was significant negative weak correlation between BMI and HF at Rec 6. WC had significant negative weak correlation with rMSSD, HF and SD1 at Rec 1, while WSR showed significant negative weak correlation with rMSSD and SD1 at Rec 6 (Table 3).

**Table 3:** Correlation between anthropometric variables and HRV indices in the moderate aerobic exercise protocol in G2. mmHg: millimeters of mercury. m: meters; kg: kilograms; mmHg: millimeters of mercury.

G2	Rest		Rec 1		Rec 2		Rec 3		Rec 4		Rec 5		Rec 6	
BMI	r	p	r	p	r	p	r	p	r	p	r	p	r	p
rMSSD	−0.18	0.35	−0.26	0.18	−0.17	0.4	−0.29	0.14	**−0.39**	**0.04**	**−0.43**	**0.02**	**−0.52**	**0.005**
HF	0.02	0.91	−0.22	0.27	−0.06	0.76	−0.29	0.14	−0.23	0.25	−0.32	0.1	**−0.48**	**0.01**
SD1	−0.18	0.35	−0.26	0.18	−0.17	0.39	−0.29	0.14	**−0.39**	**0.04**	**−0.43**	**0.02**	**−0.52**	**0.005**
**WC**	**r**	**p**	**r**	**p**	**r**	**p**	**r**	**p**	**r**	**p**	**r**	**p**	**r**	**p**
rMSSD	0.0008	0.99	**−0.39**	**0.04**	−0.24	0.21	−0.11	0.58	−0.18	0.35	−0.31	0.11	−0.33	0.09
HF	0.18	0.35	**−0.41**	**0.03**	−0.1	0.59	−0.14	0.47	−0.18	0.37	−0.33	0.09	−0.2	0.32
SD1	0.0005	0.99	**−0.39**	**0.04**	−0.25	0.21	−0.11	0.58	−0.18	0.35	−0.31	0.11	−0.33	0.09
**WSR**	**r**	**p**	**r**	**p**	**r**	**p**	**r**	**p**	**r**	**p**	**r**	**p**	**r**	**p**
rMSSD	−0.37	0.06	−0.37	0.06	−0.21	0.29	−0.18	0.37	−0.3	0.13	−0.3	0.12	**−0.45**	**0.02**
HF	−0.21	0.29	−0.29	0.13	−0.11	0.56	−0.08	0.69	−0.15	0.46	−0.28	0.15	−0.26	0.19
SD1	−0.37	0.06	−0.37	0.06	−0.21	0.29	−0.18	0.36	−0.3	0.13	−0.3	0.12	**−0.45**	**0.02**

BMI: body mass index; WC: waist circumference; WSR: waist-stature ratio; HR: heart rate; rMSSD: square root of the square mean of the differences between adjacent normal RR intervals; SD1: standard deviation of instantaneous beat-to-beat variability; HF: high frequency; G2: group with WSR between 0.45 and 0.49.

Linear regression analysis indicated that all anthropometric variables that correlated with HRV indices in the recovery from aerobic exercise in G2 had significant impact on rMSSD, SD1 and HF. BMI was the anthropometric variable that presented the greatest influence (Table 4).

**Table 4:** Linear regression between anthropometric variables and HRV indices in G2.

Models	β	95% C.I.	p	R^2^-adjusted
**1- BMI**				
rMSSD (Rec 4)	−2.893	−5.693; −0.092	0.04	0.15
rMSSD (Rec 5)	−3.214	−6.019; −0.4086	0.026	0.18
rMSSD (Rec 6)	−4.007	−6.728; −1.286	0.0057	0.27
HF (Rec 6)	−103.47	38.298; −182.51	0.0125	0.23
SD1 (Rec 4)	−2.054	0.9610; −4.037	0.04	0.159
SD1 (Rec 5)	−2.277	0.9612; −4.261	0.026	0.189
SD1 (Rec 6)	−2.844	0.9339; −4.771	0.0056	0.2787
**2- WC**				
rMSSD (Rec 1)	−87.636	41.663; −173.63	0.04	0.155
HF (Rec 1)	−1960.4	879.80; −3776.3	0.0355	0.171
SD1 (Rec 1)	−62.025	29.533; −122.9	0.0464	0.155
3- WSR				
rMSSD (Rec 6)	−359.14	144.82; −658.06	0.0206	0.204
SD1 (Rec 6)	0.2036	102.66; −466.18	0.0207	0.2036

BMI: body mass index; WC: waist circumference; WSR: waist-stature ratio; rMSSD: square root of the square mean of the differences between adjacent normal RR intervals; SD1: standard deviation of instantaneous beat-to-beat variability; HF: high frequency; r-adjusted: coefficient of determination of the percentage of variation; β: Beta; C.I.: confidence interval; G2: group with WSR between 0.45 and 0.49.

## Discussion

Considering that WSR has been applied in the assessment and treatment of obesity, we intended to draw attention to the prevention and evaluation of the autonomic recovery after submaximal and maximal aerobic exercise in healthy men with different ranges of WSR.

The main results demonstrate that the group with higher WSR achieved: (a) slower return of F to baseline values after moderate exercise; (b) delayed autonomic recovery following maximal effort and; (c) the interaction of WSR in autonomic recovery exercise is influenced by BMI. We assume that the involvement of WSR in HRV recovery is influenced by exercise intensity.

Regarding cardiorespiratory variables, it was detected that the group with moderate risk presented slightly slower return of DBP while no significant change was noted in the group with high risk. Some studies have revealed a connection between WSR and blood pressure and/or the presence of hypertension^30–32^. Taing *et al*.^31^ studied the association between the anthropometric measurements, blood pressure and hypertension in 7601 subjects aged 18 to 59 years old. They also described the relationship between WSR and BP, in which a 10 cm increase in WC was associated with an increase of 4 mmHg in SBP. They suggested that central adiposity measured by the anthropometric measurements may be a more significant determinant of blood pressure and hypertension than general adiposity.

Nevertheless, we were unable to determine any previous studies that evaluated the association of the WSR and BP dynamics following exercise. The majority of the studies assessed the association of WSR with the risk of developing hypertension or cardiovascular pathologies^4,5^.

We reported significant changes between groups regarding HR recovery from maximal effort (slower recovery in the group with moderate/high risk groups), however, there was no difference between groups in recovery of HR from moderate aerobic exercise. Previous studies exhibited that post-exercise HR recovery is related to vagal reactivation, exercise characteristics and general physical fitness level^33^, which is considered a reliable predictor of mortality^34^. In this study, both groups exhibited similar recovery of HR, which was anticipated because they were healthy and physically active individuals.

Regarding F, the group with higher WSR offered a slower return to baseline values in the recovery period after exercise. Yet, even with differences between groups, all the subjects offered physiological changes of F during exercise. This was expected as they were healthy individuals without the presence of recognized cardiopulmonary diseases.

Regarding vagal modulation, the groups with the higher WSR had delayed recovery after maximal effort. Accordingly, Koenig *et al*.^35^ found a connection between HRV and WSR when investigating the relationship of different measures of adiposity with ANS function. A total of 8538 healthy subjects of either gender submitted to HRV recordings for 24 hours were evaluated. These investigators uncovered an inverse correlation between the rMSSD index and the WSR, concluding that the HRV is related to the adiposity levels in healthy individuals.

Similar results were observed by Monteze *et al*.^36^ wherein they investigated HRV responses to postural change and, its connection with cardiovascular risk factors in 438 men with mean age of 34 years submitted to electrocardiogram recording. It was detected that resting rMSSD and HF indices had negative correlations with WSR.

In this way, our results provide strong evidence regarding the use of WSR for identifying cardiorespiratory dysfunction in maximal effort. Conversely, the BMI includes calculation based on height and mass^16^, leading to failure in providing precise information concerning adiposity measurement^37^. A previous study investigated BMI, body adiposity index and risk for type 2 diabetes mellitus. As a main finding, it was demonstrated that the body adiposity index was a better risk predictor compared to BMI. Furthermore, a very recent investigation reported interesting data. Zhao *et al*.^38^, performed a systematic review and meta-analysis and concluded that higher BMI values were related to lower mortality in patients in the intensive care unit.

A previous study reinforced the use of WSR to detect health disorders. Rodrigues *et al*.^39^, performed a cross-sectional and population-based study in 1662 individuals aged from 25 to 64 years old. The authors observed that WSR is the simplest and best obesity index to detect hypertension and metabolic syndrome compared to BMI. In this case, it was suggested that abdominal obesity measured through WSR is more reliable than overall obesity quantified with BMI.

In this circumstance, in order to evaluate the association between anthropometric variables and HRV during recovery from aerobic exercise, we performed correlation and linear regression analysis. We revealed that the group with lower WSR did not present significant association, however, we reported weak (r between 0.5 and 0.39) but significant (p < 0.05) impact of BMI on HRV indices in the final moments of recovery from moderate aerobic exercise (45 to 60 minutes). Taken together, we suggest that BMI is an important index involved in the association between WSR and autonomic recovery from aerobic exercise in healthy subjects.

Some results from this study should be highlighted. The sample used consisted of a standardized male population. Consequently, our results cannot be applied to different populations due to modifications in body composition, physical conditioning, age and sexual hormones^40^. Yet, the present study presented a standardization for the population composed of both healthy and physically active young men.

We realized that HRV was not totally recovered in 60 minutes in all groups, since it did not reach baseline values. Exercise intensity influences HRV at least during the first hour after exercise^41^. This is because during aerobic exercise, arterial baroreflex resets and metaboreflex is activated, both mechanisms are necessary to provide blood supply and oxygen to the active skeletal muscles in this situation^42^. Following exercise cessation, the metabolites removal from muscles decrease metaboreflex activation, inducing baroreflex reestablishment. Metaboreflex reduction is suggested to last around 90 minutes whereas baroreflex restoration is proposed to spend approximately 180 minutes following exercise end^43^. In this line, our study provides results concerning metaboreflex and baroreflex acting simultaneously during recovery from exercise. We are not able to isolate the involvement of baroreflex return based on our data.

Our results draw attention to the importance of cardiovascular prevention in the population within WSR values above 0.45, since we established that physically active men in this group offered slower autonomic recovery following aerobic exercise.

## Conclusion

In brief, healthy men with higher WSR offered delayed autonomic recovery after maximal effort, indicating an elevated probability of developing cardiovascular problems. The involvement of WSR in autonomic recovery following maximal effort is influenced by BMI.

## References

[1] Tchernof A, Despres JP (2013). Pathophysiology of human visceral obesity: an update. Physiol. Rev..

[2] Savva SC, Lamnisos D, Kafatos AG (2013). Predicting cardiometabolic risk: waist-to-height ratio or BMI. A meta-analysis. Diabetes Metab. Syndr. Obes..

[3] Schneider HJ (2011). Measuring abdominal obesity: effects of height on distribution of cardiometabolic risk factors risk using waist circumference and waist-to-height ratio. Diabetes Care..

[4] Guasch-Ferré M (2012). Waist-to-height ratio and cardiovascular risk factors in elderly individuals at high cardiovascular risk. PLoS ONE..

[5] Huxley R (2010). Body mass index, waist circumference and waist:hip ratio as predictors of cardiovascular risk - a review of the literature. Eur. J. Clin. Nutr..

[6] Ashwell M, Gunn P, Gibson S (2012). Waist-to-height ratio is a better screening tool than waist circumference and BMI for adult cardiometabolic risk factors: systematic review and meta-analysis. Obes. Rev..

[7] Hsieh SD, Muto T (2005). The superiority of waist-to-height ratio as an anthropometric index to evaluate clustering of coronary risk factors among non-obese men and women. Prev. Med..

[8] Yang XY (2016). Body mass index, waist circumference and waist-to-height ratio associated with the incidence of type 2 diabetes mellitus: a cohort study. Zhonghua Yu Fang Yi Xue Za Zhi..

[9] Vidal MM (2015). Anthropometric indicators of obesity as predictors of cardiovascular risk in the elderly. Nutr. Hosp..

[10] Dantas EMS, Pinto CJ, Freitas RPA, Medeiros ACQ (2015). Agreement in cardiovascular risk rating based on anthropometric parameters. Einstein..

[11] Indumathy J (2015). Association of sympathovagal imbalance with obesity indices, and abnormal metabolic biomarkers and cardiovascular parameters. Obes. Res. Clin. Pract..

[12] Task F (1996). of the European Society of Cardiology and the North American Society of Pacing and Electrophysiology. Heart rate variability: standards of measurement, physiological interpretation and clinical use. Circulation..

[13] Xhyheri B, Manfrini O, Mazzolini M, Pizzi C, Bugiardini R (2012). Heart rate variability today. Prog. Cardiovasc. Dis..

[14] Peçanha T, Silva-Júnior ND, Forjaz CL (2014). Heart rate recovery: autonomic determinants, methods of assessment and association with mortality and cardiovascular diseases. Clin. Physiol. Funct. I..

[15] Pardini R (2001). Validation of the international questionnaire on the level of physical activity (IPAQ -version6): a pilot study in young Brazilian adults. Rev. Bras. Ciên. e Mov..

[16] Lohman, T. G., Roche, A. F. & Martorelli, R. *Anthropometric Standardization Reference Manual*. Champaign: Human Kinetics Books (1988).

[17] Ashwell M, Hsieh SD (2005). Six reasons why the waist-to-height ratio is a rapid and effective global indicator for health risks of obesity and how its use could simplify the international public health message on obesity. Int. J. of Food Sci. Nutr..

[18] Gomes, R. L., Gonzaga, L. A., Vanderlei, L. C. & Valenti, V. E. The effects of musical auditory stimulation on cardiorespiratory variables after aerobic exercise. *Science Sports*. Epub ahead of print (2018).

[19] Gomes RL, Vanderlei LCM, Garner DM, Vanderlei FM, Valenti V (2017). Higuchi Fractal Analysis of Heart Rate Variability is Sensitive during Recovery from Exercise in Physically Active Men. Medical Express..

[20] Conconi F, Ferrari M, Ziglio PG, Droghetti P, Codeca L (1982). Determination of the anaerobic threshold by a non-invasive yeld test in runners. J. Appl. Physiol..

[21] Araujo, C. G. S. *ACSM Manual for Exercise and Exercise Exercise Testing*. 5ª Ed. Revinter. Rio de Janeiro - RJ (2000).

[22] Cambri LT, Foza V, Nakamura FY, Oliveira FR (2006). Heart rate and the identification of the metabolic transition points in a treadmill. R. da Educação Física/UEM Maringá..

[23] Borg, G. *Borg’s Scales for Pain and Perceived Effort*. (Manole, 2000).

[24] Vanderlei LCM, Silva RA, Pastre CM, Azevedo FM, Godoy MF (2008). Comparison of the Polar S810i monitor and the ECG for the analysis of heart rate variability in the time and frequency domains. Braz. J. Med. Biol. Res..

[25] Martiniano EC (2018). Musical auditory stimulus acutely influences heart rate dynamic responses to medication in subjects with well-controlled hypertension. Sci. Rep..

[26] Gonzaga LA, Vanderlei LCM, Gomes RL, Valenti VE (2017). Caffeine affects autonomic control of heart rate and blood pressure recovery after aerobic exercise in young adults: a crossover study. Sci. Rep..

[27] Hoshi RA (2017). Temporal sequence of recovery-related events following maximal exercise assessed by heart rate variability and blood lactate concentration. Clin. Physiol. Funct. Imaging..

[28] Bastos FN (2012). Effects of cold water immersion and active recovery on post-exercise heart rate variability. Int. J. Sports Med..

[29] Vanderlei LCM, Pastre CM, Hoshi RA, Carvalho TD, Godoy MF (2009). Basics of heart rate variability and its clinical applicability. Rev. Bras. Cir. Cardiovasc..

[30] Zhou Z, Hu D, Chen J (2009). Association between obesity indices and blood pressure or hypertension: which index is the best?. Public Health Nutr..

[31] Taing KY, Farkouh ME, Moineddin R, Tu JV, Jha P (2016). Age and sex-specific associations of anthropometric measures of adiposity with blood pressure and hypertension in India: a cross-sectional study. BMC Cardiovasc. Disord..

[32] Ononamadu CJ (2017). Comparative analysis of anthropometric indices of obesity as correlates and potential predictors of risk for hypertension and prehypertension in a population in Nigeria. Cardiovasc. J. Afr..

[33] Belli JFC, Bacal F, Bocchi EA, Guimarães GV (2011). Ergorreflex behavior in heart failure. Arq. Bras. Cardiol..

[34] Danieli A (2014). Resting heart rate variability and heart rate recovery after submaximal exercise. Clin. Auton. Res..

[35] Koenig J (2015). Association Strength of Three Adiposity Measures with Autonomic Nervous System Function in Apparently Healthy Employees. J. Nutr. Health Aging..

[36] Monteze, N. M. *et al*. Heart Rate Variability in Shift Workers: Responses to Orthostatism and Relationships with Anthropometry, Body Composition, andBlood Pressure. *BioMed. Res Int*. Article ID329057 (2015). 10.1155/2015/329057 PMC4606218 26495293

[37] Arrebola-Moreno AL (2014). Body mass index and myocardium at risk in patients with acute coronary syndrome. Rev Clin Esp..

[38] Zhao Y, Li Z, Yang T, Wang M, Xi X (2018). Is body mass index associated with outcomes of mechanically ventilated adult patients in intensive critical units? A systematic review and meta-analysis. PLoS One..

[39] Rodrigues SL, Baldo MP, Mill JG (2010). Association of waist-stature ratio with hypertension and metabolic syndrome: population-based study. Arq. Bras. Cardiol..

[40] Sydó N (2014). Relationship between exercise heart rate and age in men vs. women. Mayo Clin. Proc..

[41] Michael S, Jay O, Halaki M, Graham K, Davis GM (2016). Submaximal exercise intensity modulates acute post-exercise heart rate variability. Eur. J. Appl. Physiol..

[42] Kaufman MP (2012). The exercise pressor reflex in animals. Exp. Physiol..

[43] Shoemaker JK, Badrov MB, Al-Khazraji BK, Jackson DN (2015). Neural Control of Vascular Function in Skeletal Muscle. Compr. Physiol..

